# Calcination Method Synthesis of SnO_2_/g-C_3_N_4_ Composites for a High-Performance Ethanol Gas Sensing Application

**DOI:** 10.3390/nano7050098

**Published:** 2017-04-29

**Authors:** Jianliang Cao, Cong Qin, Yan Wang, Bo Zhang, Yuxiao Gong, Huoli Zhang, Guang Sun, Hari Bala, Zhanying Zhang

**Affiliations:** 1 School of Chemistry and Chemical Engineering, Henan Polytechnic University, Jiaozuo 454000, China; caojianliang@hpu.edu.cn (J.C.); qincongxy@163.com (C.Q.); zhb@hpu.edu.cn (B.Z.); gyx201311@163.com (Y.G.); zhanghuoli@hpu.edu.cn (H.Z.); mcsunguang@hpu.edu.cn (G.S.); hari@hpu.edu.cn (H.B.); 2 School of Safety Science and Engineering, State Key Laboratory Cultivation Base for Gas Geology and Gas Control, Henan Polytechnic University, Jiaozuo 454000, China

**Keywords:** graphitic carbon nitride, SnO_2_, calcination method, SnO_2_/g-C_3_N_4_ composite, ethanol gas sensing

## Abstract

The SnO_2_/g-C_3_N_4_ composites were synthesized via a facile calcination method by using SnCl_4_·5H_2_O and urea as the precursor. The structure and morphology of the as-synthesized composites were characterized by the techniques of X-ray diffraction (XRD), the field-emission scanning electron microscopy and transmission electron microscopy (SEM and TEM), energy dispersive spectrometry (EDS), thermal gravity and differential thermal analysis (TG-DTA), and N_2_-sorption. The analysis results indicated that the as-synthesized samples possess the two dimensional structure. Additionally, the SnO_2_ nanoparticles were highly dispersed on the surface of the g-C_3_N_4_nanosheets. The gas-sensing performance of the as-synthesized composites for different gases was tested. Moreover, the composite with 7 wt % g-C_3_N_4_ content (SnO_2_/g-C_3_N_4_-7) SnO_2_/g-C_3_N_4_-7 exhibits an admirable gas-sensing property to ethanol, which possesses a higher response and better selectivity than that of the pure SnO_2_-based sensor. The high surface area of the SnO_2_/g-C_3_N_4_ composite and the good electronic characteristics of the two dimensional graphitic carbon nitride are in favor of the elevated gas-sensing property.

## 1. Introduction

In recent years, poisonous and harmful gases of industrial production have frequently leaked. Meanwhile, organic poisonous gases, such as methylbenzene and formaldehyde, volatilize from furniture and newly-decorated houses. As a result, it severely threatens the health of mankind [1,2,3,4]. Therefore, there is the need for an effective and necessary method for detecting the vaporable substances. In the past several years, considerable attentions have been dedicated to metal-oxide semiconductor (MOS) material-based gas sensors. Various metal-oxide semiconductors (MOS), such as SnO_2_ [5], ZnO [6], CuO [7], α-Fe_2_O_3_ [8], Co_3_O_4_ [9], MnO_2_ [10], WO_3_ [11], In_2_O_3_ [12], and NiO [13], are widely used as gas sensors. These gas sensors exhibited unique performances, including a low-cost, small size, and fast response and recovery time. SnO_2_, a typical n-type metal-oxide semiconductor with a rutile crystalline structure, is widely used to detect different kinds of gases such as ethanol [14], formaldehyde [15], acetone [16], nitrogen dioxide [17], etc. These are all due to their remarkable characteristics such as their good chemical and physical stability, non-pollution property, low-energy feature, use of simple preparation, and so on.

The most remarkable characteristic of SnO_2_ is that the resistance varies when it is exposed to different types of target gases. As a rule, the resistance value of SnO_2_ decreases when it is exposed in reducing gases, and conversely, it increases under the oxidizing gases. As we all know, there are many oxygen vacancies of SnO_2_ which are in favor of the process of gas adsorption. In general, it has a better gas-sensing performance with a higher concentration of oxygen vacancies. Nonetheless, there are some significant defects which restricts its application in gas sensors. For instance, a high working temperature, long response and recovery time, high resistance, and easy agglomeration draw the attention of researchers. In order to overcome these shortcomings, it is necessary to explore an electroconductive intermediate to support SnO_2_ nanoparticles, in order to improve the electrical conductivity and dispersity. Therefore, many two-dimensional (2D) carbon materials are widely used as intermediates [18,19,20]. Graphene, a unilaminar sp^2^-hybridized carbon atoms configuration, exhibits an excellent performance, including a large specific surface area, better electronic conductivity, and superior stability. On account of these advantages, graphene and reduced graphene oxide are widely used as gas-sensing materials to detect different kinds of gases. Different metal-oxide decorated graphene nanocomposite gas sensors were reported and exhibited outstanding gas-sensing performances [21,22,23,24]. However, the process of preparing GO and r-GO is complicated and high-cost. Hence, it is necessary to explore a novel 2D structure material with graphene.

Recently, graphitic carbon nitride (g-C_3_N_4_) has attracted increasing attention due to its high photology and chemical stability, facile preparation, high specific surface area, and nontoxicity [25,26,27,28]. In general, g-C_3_N_4_ is readily synthesized by calcining abundant nitrogen-rich precursors such as melamine, dicyandiamide, and urea. It has been widely used in fields such as photocatalysis, degradation, and energy storage materials [29,30,31]. Until now, there are few reports on the application of g-C_3_N_4_ in the field of sensors. Zeng et al. have successfully prepared the α-Fe_2_O_3_/g-C_3_N_4_ nanocomposite through a facile refluxing method for the cataluminescence sensing of H_2_S [32]. Zhang et al. have developed a novel fluorescence sensor based on a g-C_3_N_4_/MnO_2_ sandwich nanocomposite for the rapid and selective sensing of glutathione [33]. Moreover, CeO_2_/g-C_3_N_4_ composites with high photocatalytic activity have been synthesized successfully [34]. To the best of the authors’ knowledge, SnO_2_/g-C_3_N_4-_based sensors have yet not been reported in the literature.

In this work, we report a facile calcination approach to synthesize different mass ratios of SnO_2_/g-C_3_N_4_ composites for ethanol sensing. The gas-sensing properties, including the selectivity, stability, and sensitivity, of SnO_2_/g-C_3_N_4_ to ethanol, were investigated. As a result, the SnO_2_/g-C_3_N_4_ composite-based sensor exhibited a higher response value and better selectivity to ethanol than that of the pure SnO_2_ nanoparticle-based sensor. The mechanism of the as-prepared sample gas-sensing to ethanol was discussed, in detail.

## 2. Results and Discussion

### 2.1. Sample Characterization

**Figure 1:**
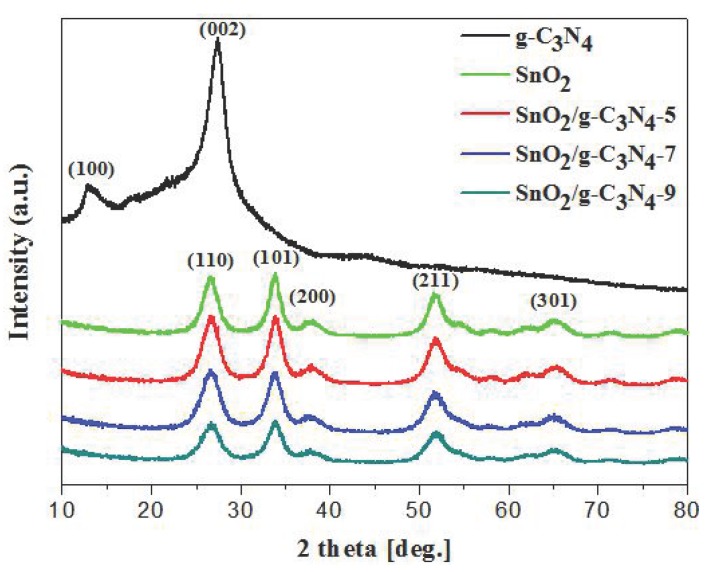
XRD patterns of the g-C_3_N_4_, SnO_2_, SnO_2_/g-C_3_N_4_-5, SnO_2_/g-C_3_N_4_-7, and SnO_2_/g-C_3_N_4_-9 composites.

Figure 1 shows the XRD patterns of the as-prepared pure SnO_2_ particles and SnO_2_/g-C_3_N_4_ composites. As demonstrated by the curves, five distinct diffraction peaks around 26.6°, 33.8°, 37.9°, 51.7°, and 65.9° can be seen, which correspond to (110), (101), (200), (211), and (301) planes of the tetragonal rutile structure SnO_2_ (JCPDS Card No. 41-1445), respectively. However, the diffraction peaks of g-C_3_N_4_ in the SnO_2_/g-C_3_N_4_ composites are not observed in the curves. This may be due to the relatively small content of g-C_3_N_4_ in the composites. Another reason is that the peak around 27.5° of g-C_3_N_4_ is overlapped by the peak around 26.6° of SnO_2_.

**Figure 2:**
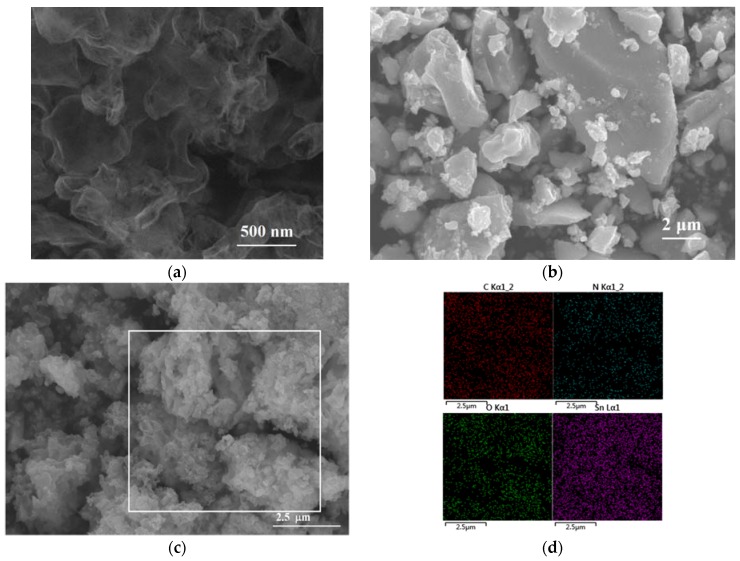
SEM images of (**a**) g-C_3_N_4_, (**b**) SnO_2_ and (**c**) SnO_2_/g-C_3_N_4_-7 composite, (**d**) the EDS mappings of C, N, O, and Sn elements related to the selected area in (**c**).

The SEM images of the g-C_3_N_4_, SnO_2_, and SnO_2_/g-C_3_N_4_-7 composites are displayed in Figure 2. Figure 2a displays the SEM image of the g-C_3_N_4_ sample. We can see many wrinkles on the edge of the thin layers, which represents the nanosheet structure. Figure 2b shows many SnO_2_ particles agglomerated together with different sizes. This phenomenon indicates that pure SnO_2_ particles are easy to agglomerate, which is harmful to the process of gas adsorption. As shown in Figure 2c, many nanoparticles are attached to the g-C_3_N_4_ nanosheet. This could be beneficial to improving the gas-sensing properties. Figure 2d illustrates the typical EDS mappings of the SnO_2_/g-C_3_N_4_-7 composite recorded from the surface area that is observed in Figure 2c, in which four elements of C, N, Sn, and O are concurrently existent. It can be concluded that SnO_2_ and g-C_3_N_4_ are coexistent in the composite.

**Figure 3:**
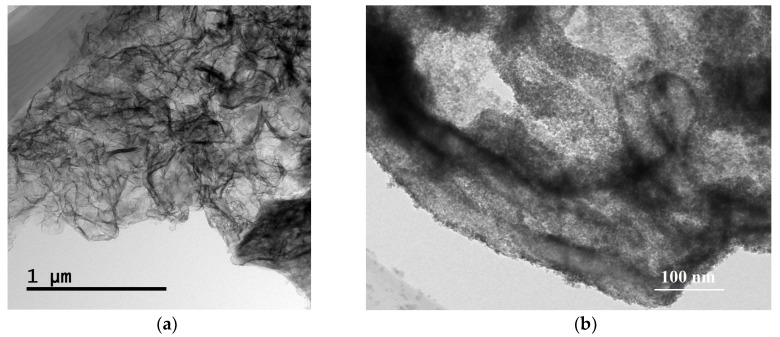
TEM images of (**a**) g-C_3_N_4_ and (**b**) SnO_2_/g-C_3_N_4_-7 composite.

Figure 3 shows the TEM images of g-C_3_N_4_ and the SnO_2_/g-C_3_N_4_-7 composite. We can clearly see from Figure 3a that there are a lot of folds which seem like floccules, clearly demonstrating the existence of the g-C_3_N_4_ nanosheet. The lines represent the stacked rolled edges of the nanosheet structure. As seen from Figure 3b, the SnO_2_ particles are highly dispersed on the surface of the g-C_3_N_4_ nanosheet. This overcame the disadvantage of an easy agglomeration of SnO_2_ particles and further enhanced the specific surface area. Therefore, this test identifies it as an excellent candidate for gas-sensing materials.

**Figure 4:**
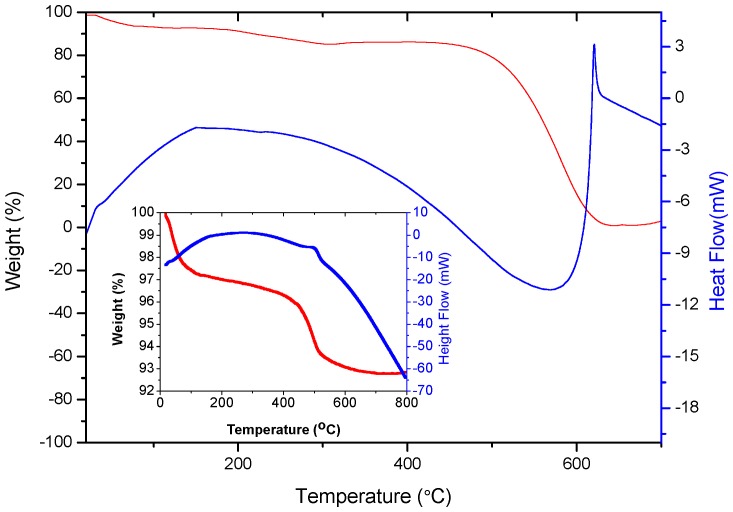
TG-DTA profiles of the g-C_3_N_4_ and SnO_2_/g-C_3_N_4_-7 composite.

TG-DTA was carried out to reveal the weight change situation of g-C_3_N_4_ and SnO_2_/g-C_3_N_4_-7. The temperature range is from room temperature to 700 °C, and the heating rate is 10 °C·min^−1^. As is shown in Figure 4, the red and blue lines correspond to weight and heat flow curves, respectively. The first agravity peak is between 100 °C and 300 °C, which is due to the desorption of moisture and the solvent. The second agravity peak is between 400°C and 600°C, which is due to the combustion of g-C_3_N_4_ in air. The inset in Figure 4 is the TG-DTA profiles of SnO_2_/g-C_3_N_4_-7. The agravic peak below 400°C is due to the desorption of solvent, and the remanent content of the composite is 92% after the combustion of g-C_3_N_4_. This result demonstrated that g-C_3_N_4_ was not decomposed at the optimum temperature of 300°C in the process of testing the gas-sensing properties.

**Figure 5:**
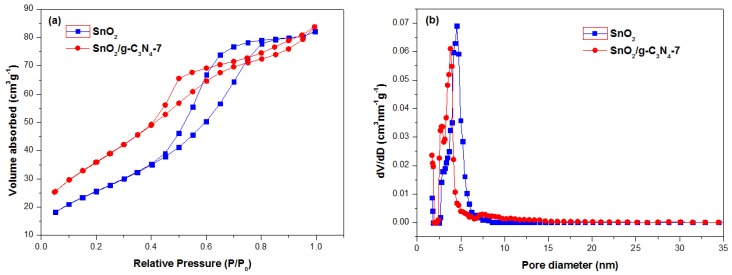
(**a**) N_2_ adsorption-desorption isotherms and (**b**) the corresponding pore size distribution curves of the SnO_2_ and SnO_2_/g-C_3_N_4_-7 composite.

Figure 5 depicts the N_2_ adsorption-desorption isotherms and the corresponding pore size distribution of the as-prepared SnO_2_ and SnO_2_/g-C_3_N_4_-7 samples. It can be seen from Figure 5a that the isotherms of the two samples show type IV, which are the typical characteristics of mesoporous materials according to the IUPAC. The well-defined hysteresis loop belonging to the H_3_-type clearly indicates the existence of an aggregation of the laminated structure with a narrow slit formed by the g-C_3_N_4_ and SnO_2_ nanoparticles. Figure 5b displays the corresponding pore size distribution of the two samples. It can be clearly seen that the pore diameter of SnO_2_ and SnO_2_/g-C_3_N_4_ are relatively small and that the majority concentrate upon 4.54 nm and 3.79 nm, respectively, according to the DFT method. The BET calculated results show that the specific surface area of the SnO_2_ and SnO_2_/g-C_3_N_4_-7 samples are 94.3 m^2^·g^−1^ and 132.5 m^2^·g^−1^, respectively. The specific surface area of the as-prepared composite has been significantly improved, which could be in favor of enhancing the gas-sensing properties.

### 2.2. Gas-Sensing Performance

**Figure 6:**
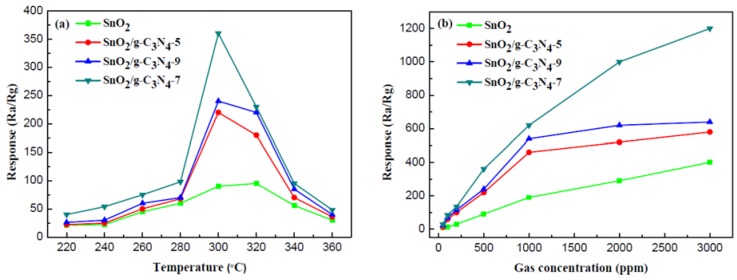
(**a**) Response values of the sensors based on SnO_2_, SnO_2_/g-C_3_N_4_-5, SnO_2_/g-C_3_N_4_-7, and SnO_2_/g-C_3_N_4_-9 to 500 ppm ethanol as a function of the operating temperature; (**b**) the responses of sensors (SnO_2_, SnO_2_/g-C_3_N_4_-5, SnO_2_/g-C_3_N_4_-7, and SnO_2_/g-C_3_N_4_-9) operated at 300 °C versus different concentrations of ethanol.

The gas-sensing properties of the as-prepared samples in relation ethanol were investigated, in detail. Figure 6a shows the response values of pure SnO_2_ and SnO_2_/g-C_3_N_4_-based sensors to 500 ppm ethanol at different operating temperatures. It can be clearly observed from the line chart of the three SnO_2_/g-C_3_N_4_ samples that the response values increase with the increase of the operating temperature under 300 °C. However, the response values decrease when the temperature is above 300 °C. The maximum response of SnO_2_/g-C_3_N_4_-7 is 360 at 300 °C. In stark contrast, the maximum response of the pure SnO_2_ is only 95 at 320°C. This phenomenon can be explained by the fact that the SnO_2_/g-C_3_N_4_-7-based sensor can tend to the balance between the speeds of chemical adsorption and desorption at a lower temperature, and reach a higher response than that of the pure SnO_2_ sensor. This result indicates that the SnO_2_/g-C_3_N_4_-based sensor has a great influence and enhances the gas-sensing properties for ethanol. It reaches the maximum response when the mass percentage of g-C_3_N_4_ in the composites is 7%. The specific surface area of the SnO_2_, SnO_2_/g-C_3_N_4_-5, SnO_2_/g-C_3_N_4_-7, and SnO_2_/g-C_3_N_4_-9 composites is 94.3 m^2^·g^−1^, 113.8 m^2^·g^−1^, 132.5 m^2^·g^−1^, and 122.2 m^2^·g^−1^, respectively. When the g-C_3_N_4_ content in the composites exceeds a certain value (e.g., 7 wt % in this work), it may form the connection of bulk. As a result, the specific surface area of the composite will decrease and there will be a reduced number of active sites for the adsorption of oxygen and ethanol gas, leading to the degradation of gas-sensing properties. Consequently, the gas sensor performance increases at first and decreases when the g-C_3_N_4_ content in the composites increases. A suitable amount of g-C_3_N_4_ in the composite is beneficial to the dispersity, and a preferable heterojunctional structure can be formed in the interface region between 2D g-C_3_N_4_ and SnO_2_. A high content of 2D g-C_3_N_4_ may lead to the connection of the g-C_3_N_4_ nanosheets, which could form micro electric bridges on the surface. The micro electric bridges may result in the semiconductor’s resistance being reduced, causing a reduction in the gas sensor performance. Figure 6b displays the response values of the four samples at 300 °C to different concentrations of ethanol. As shown in the line chart, the response values increase with the increasing ethanol concentrations. The slope of the curves increases rapidly when the concentration range of ethanol is from 50 ppm to 500 ppm. However, it increases slowly gradually with an increasing concentration in the range of 500–3000 ppm. Furthermore, the responses of the SnO_2_/g-C_3_N_4_ sensors are much higher than that of pure SnO_2_. It can be concluded that the adsorption of ethanol has approached the saturation value when the concentration reaches 3000 ppm.

**Figure 7:**
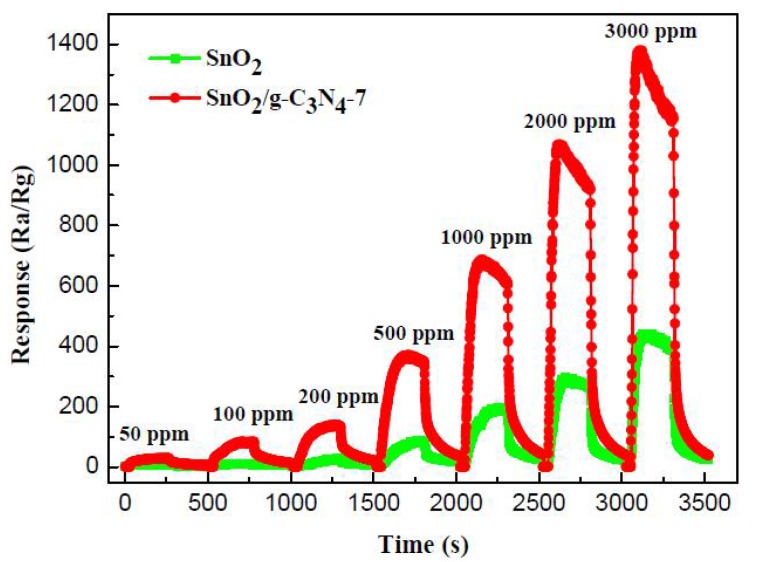
Real time response curves of the pure SnO_2_ and SnO_2_/g-C_3_N_4_-7 to ethanol in the range of 50–3000 ppm.

Figure 7 displays the real time successive response-recover curves of the pure SnO_2_ and SnO_2_/g-C_3_N_4_-7 to 500 ppm ethanol in the range of 50–3000 ppm at 300 °C. As shown by the curves, the response values of both sensors increase with the increasing concentration. The response value of the SnO_2_/g-C_3_N_4_-7-based sensor is much higher than that of the pure SnO_2_-based sensor to the same concentration of ethanol. The gas-sensing properties of the composites enhanced a lot, which is consistent with the expected.

**Figure 8:**
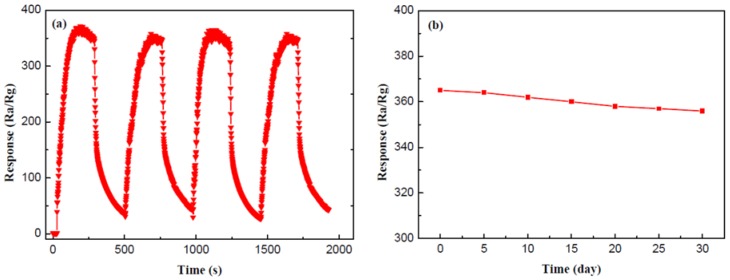
(**a**) Repeatability and (**b**) stability measurements of the SnO_2_/g-C_3_N_4_-7-based sensor to 500 ppm ethanol at 300 °C.

The repeatability and stability are both crucial criteria to measure the gas-sensing properties. Figure 8a reveals the repeatability of the SnO_2_/g-C_3_N_4_-7 sensor to 500 ppm ethanol at 300 °C. As shown by the curves, the response values of the four response-recovery cycles are almost maintained at about 360. It can be concluded that the composite sensor has an admirable repeatability for ethanol gas sensing. A durable response value was measured to explore the stability of the SnO_2_/g-C_3_N_4_-7 sensor. Figure 8b displays the test result every five days, and the response values to 500 ppm ethanol at 300 °C are maintained at around 360. Therefore, we may safely draw the conclusion that the SnO_2_/g-C_3_N_4_-7-based sensor has an unexceptionable stability for ethanol gas sensing.

**Figure 9:**
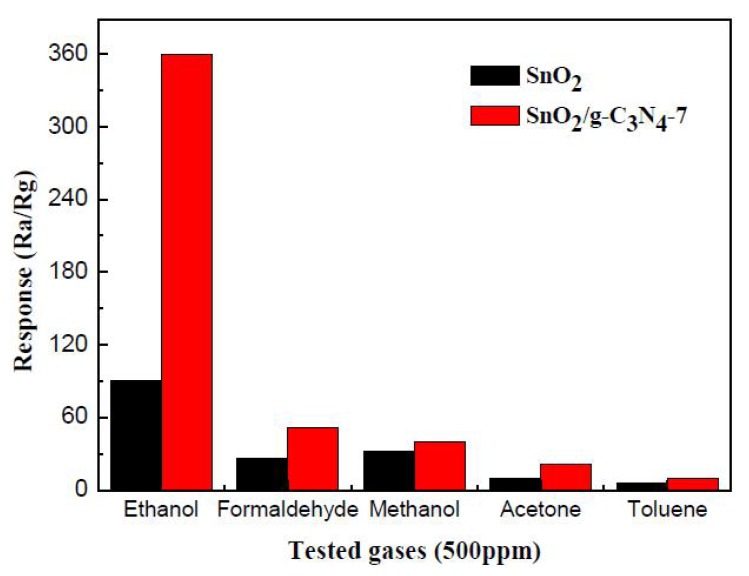
Responses of SnO_2_ and SnO_2_/g-C_3_N_4_-7-based sensors to 500 ppm different reducing gases at 300 °C.

It is generally known that selectivity is another key criterion of gas sensors. Figure 9 summarizes the selectivity test results of the pure SnO_2_ and SnO_2_/g-C_3_N_4_-7 sensors to five different gases of 500 ppm, including methanol, ethanol, toluene, formaldehyde, and acetone. It can be seen that the SnO_2_/g-C_3_N_4_-7 sensor exhibits a higher response to ethanol than to other gases compared to the pure SnO_2_ sensor. The higher responses to ethanol may be because ethanol is more likely to lose electrons in the process of the redox reaction with the absorbed oxygen and hydroxyl group (−OH) and is much easier to oxidize at the optimum operating temperature [35]. 

The gas-sensing performance of different sensing materials when using ethanol is listed in Table 1. As can be seen from Table 1, the response values of Fe_2_O_3_ nanoparticles coated with SnO_2_ nanowires [14], α-Fe_2_O_3_/g-C_3_N_4_ [35], RGO-SnO_2_ [36], and Au/SnO_2_ [37] were 31.18, 7.76, 70.4, and 18, respectively. In this work, the response value of SnO_2_/g-C_3_N_4_-7 to 100 ppm of ethanol vapor was 85 at 300 °C. Therefore, the SnO_2_/g-C_3_N_4_ composites show an excellent sensing property to ethanol vapor, and thus have a great potential application.

**Figure 10:**
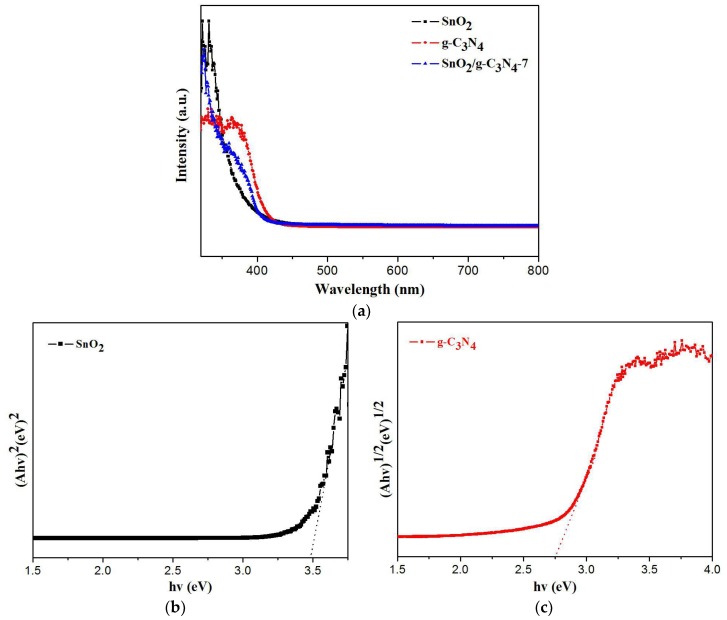
(**a**) UV-vis diffuse reflectance spectra of SnO_2_, g-C_3_N_4_, and SnO_2_/g-C_3_N_4_-7 composites, (**b**) plot of (Ahv)^2^ versus energy (hv) for the band gap energy of SnO_2_, (**c**) plot of (*Ahν*)^1/2^ versus energy (*hν*) for the band gap energy of g-C_3_N_4_.

As displayed in Figure 10a, the absorption edge of SnO_2_, SnO_2_/g-C_3_N_4_-7 and g-C_3_N_4_ are around 377 nm, 441 nm, and 455 nm, respectively. This result can be ascribed to the heterojunction structure between SnO_2_ and g-C_3_N_4_. The band gap energies of SnO_2_ and g-C_3_N_4_ can be estimated according to the equation (*A*h*ν* = *k*(*hν*-*E*g)*^n^*^/2^). In this equation, *n* is determined by the type of optical transition of a semiconductor. The value for g-C_3_N_4_ and SnO_2_ is 4 and 1, respectively. *A*, *ν*, *E*g, *k* and *h* are the absorption coefficient, light frequency, Planck constant, band gap and a constant, respectively. The diagrams are shown in Figure 10b,c. The *E*g of SnO_2_/g-C_3_N_4_-7 should be roughly calculated according to the equation (*E*g = 1240/*λ*) because of the uncertain type of optical transition. As a result, the *E*g of SnO_2_, g-C_3_N_4_, and SnO_2_/g-C_3_N_4_-7 are 3.49 eV, 2.75 eV, and 2.81 eV, respectively.

The sensing mechanism of the SnO_2_/g-C_3_N_4_ composite towards ethanol gas need to be further investigated. When the sensor was exposed in air, oxygen molecules were adsorbed on the surface of SnO_2_ and capture electrons from the conduction band of SnO_2_. Then oxygen molecules were ionized to O^2−^, O^−^, and O_2_^−^, and the formation of depletion layers led to the increase in the resistance of the composite sensor. However, when the sensor was exposed to the ethanol gas, the ethanol molecules proceeded oxidation and the reduction reaction with oxygen ions absorbed on the surface of the sensor. Concurrently, the ethanol molecules were oxidized into acetaldehyde and eventually turned into carbon dioxide and water [24]. As a result, the trapped electrons were released back to the depletion layer of the sensing film, resulting in the decrease in the resistance of the composite-based sensor. The SnO_2_/g-C_3_N_4_ composite exhibited more preferable gas-sensing properties than that of pure SnO_2_. This is mainly due to the high specific surface area and the interaction between g-C_3_N_4_ and SnO_2_. The presence of g-C_3_N_4_ can prevent the aggregation of SnO_2_ particles to form a high surface approachability structure, leading to the promotion of the adsorption and diffusion process of ethanol molecules. In this composite, the s-triazine structure g-C_3_N_4_ sheet substrate can provide more active sites to adsorb O_2_ molecules. The elevated gas-sensing properties may also be due to the interactions between Sn and g-C_3_N_4_, and the heterojunction of the interface region between g-C_3_N_4_ and SnO_2_. The electrical property at the heterojunction changes when ethanol gas molecules pass through the interface region between g-C_3_N_4_ and SnO_2_. Both SnO_2_ and g-C_3_N_4_ are n-type semiconductors. The band gaps are 3.71 eV and 2.7 eV, respectively. The conduction band level of g-C_3_N_4_ is more negative than SnO_2_. When SnO_2_ and g-C_3_N_4_ were combined, they formed a heterojunction structure. The electrons will thus inflow from the conduction band of g-C_3_N_4_ to the conduction band of SnO_2_, leading to a higher potential barrier. As a result, the electrons and holes are separated [38]. Meanwhile, the heterojunction structure may suppress the recombination of the electron-hole and urge electrons to quickly transfer from the ethanol vapor to the surface of SnO_2_/g-C_3_N_4_. Therefore, this leads to a higher response because of the increased conductivity of the heterojunction structure [35]. This co-adjacent retiform structure could provide more accesses for the gas adsorption and diffusion between SnO_2_ and ethanol molecules.

(1) $$O_{2\;}+e^{-}\to O_{2}^{-}$$

(2) $$2{\mathrm{CH}}_{3}{\mathrm{CH}}_{2}\mathrm{OH}+O_{2}^{-}\to 2{\mathrm{CH}}_{3}\mathrm{CHO}+2H_{2}O+e^{-}$$

(3) $$2{\mathrm{CH}}_{3}\mathrm{CHO}+5O_{2}^{-}\to 4{\mathrm{CO}}_{2}+4H_{2}O+5e^{-}$$

## 3. Materials and Methods 

### 3.1. Chemicals

Urea and Tin (IV) chloride pentahydrate (SnCl_4_·5H_2_O, 99.0%) were purchased from Macklin Biochemical Co., Ltd. (Shanghai, China). All chemicals were used as received, without further purification.

### 3.2. Preparation of g-C_3_N_4_

Graphitic carbon nitride (g-C_3_N_4_) wasdirectly synthesized by the pyrolysis of urea in the muffle furnace (Luoyang Shenjia Kiln Co., Ltd., Luoyang, Henan, China). A total of 20 g urea was put into an alumina crucible with a cover and then warmed to a temperature of 250 °C within 110 min, before being kept at 250 °C for 1 h. Then, the temperature was increased to 350 °C within 50 min and kept at 350 °C for 2 h. Finally, temperature rose to 550 °C within 100 min and was kept at 550 °C for another 2 h. The heating rate of the whole reaction was 2 °C·min^−1^. The resulting yellow powder was collected.

### 3.3. Synthesis of the SnO_2_/g-C_3_N_4_Composite

SnO_2_/g-C_3_N_4_ composites were synthesized through a facile calcination method. In a typical preparation process, a certain amount g-C_3_N_4_ was dissolved in 100 mL H_2_O and 2.09 g SnCl_4_·5H_2_O was added into the dispersed suspension with ultrasonic treatment for 2 h. Then, the mixed solution was transferred into an alumina crucible and put it into the muffle furnace. It was heated to 400 °C for 2 h. Finally, the resulting product was ground to powder. According to this method, the different mass ratios of the SnO_2_/g-C_3_N_4_ composites were synthesized and marked as SnO_2_/g-C_3_N_4_-5, SnO_2_/g-C_3_N_4_-7, and SnO_2_/g-C_3_N_4_-9. For comparison, the same method was used to synthesize the pure SnO_2_ particles with the absence of g-C_3_N_4_.

### 3.4. Characterizations

The samples were characterized by X-ray diffraction (XRD, Bruker-AXS D8, Bruker, Madison, WI, USA) with Cu Kα radiation at 40 kV and 25 mA. The structure and morphology of the samples were observed by field-emission scanning electron microscopy (FESEM, Quanta™ 250 FEG) (FEI, Eindhoven, The Netherlands). Transmission electron microscopy (TEM) analysis was performed on a JEOL JEM-2100 microscope (JEOL, Tokyo, Japan) operating at 200 kV. The thermal gravity and differential thermal analysis (TG-DTA) was carried on TA-SDT Q600 (TA, New Castle, DE, USA) at a heating rate 10 °C·min^−^^1^ under an air atmosphere. Nitrogen adsorption-desorption isotherms were obtained on a Quantachrome Autosorb-iQ sorption analyzer (Quantachrome, Boynton Beach, FL, USA). Before carrying out the measurement, the samples were degassed for more than 6 h at 300 °C.

### 3.5. Gas-Sensing Test

**Figure 11:**
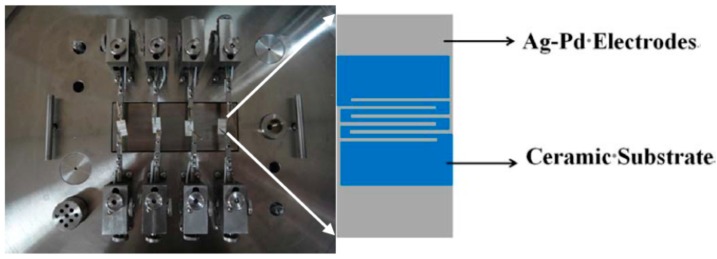
The internal structure diagram of the CGS-4TPS gas-sensing test system and the structure of the substrate.

The gas-sensing performance of the as-synthesized samples when using ethanol was tested by using the intelligent gas-sensing analysis system of CGS-4TPS (Beijing Elite Co., Ltd., Beijing, China). Figure 11 shows a brief schematic diagram of the device. The gas sensors were prepared according to the usual way. A small amount of the as-prepared sample was fully ground in agate mortar with a few drops ethanol, which served as the agglomerant to form starchiness. Afterwards, the pastes were equably spread on the ceramic substrate (13.4 mm × 7 mm) with interdigitated Ag-Pd electrodes to form the thin film. Before carrying out the test, the substrate was aged at 180 °C for 24 h to improve the stability and repeatability of the gas sensors. The response of the sensors was defined as the ratio of R_a_/R_g_, where R_a_ and R_g_ were the resistance of sensor in air and in the target gas, respectively. 

## 4. Conclusions

In this study, the SnO_2_/g-C_3_N_4_ composites with a high surface area (132.5 m^2^·g^−1^) were synthesized via a facile calcination method by using SnCl_4_·5H_2_O and urea as the precursor. The SnO_2_ particles were highly distributed on the g-C_3_N_4_ nanosheets. The gas-sensing properties of the SnO_2_/g-C_3_N_4_-7 composite-based sensor exhibited preferable results when compared to the pure SnO_2_, including the sensitivity and selectivity. Considering the easy-preparation process, the SnO_2_/g-C_3_N_4_ composite could be a promising candidate for high-performance ethanol gas-sensing applications.

## Figures and Tables

**Table 1:** Gas-sensing performance comparison of various gas sensors toward ethanol.

Sensing Materials	Ethanol Concentration (ppm)	Temperature (°C)	Response (R_a_/R_g_)	Ref.
Fe_2_O_3_-SnO_2_	100	300	31.18	[14]
α-Fe_2_O_3_/g-C_3_N_4_	100	340	7.76	[35]
RGO-SnO_2_	100	300	70.4	[36]
Au/SnO_2_	100	340	18	[37]
SnO_2_/g-C_3_N_4_	100	300	85	this work
